# Evaluation of postcontrast images of intracranial tumors at 7T and 3T MRI: An intra‐individual comparison study

**DOI:** 10.1111/cns.14036

**Published:** 2022-12-05

**Authors:** Kun Cheng, Qi Duan, Jianxing Hu, Chenxi Li, Xiaoxiao Ma, Xiangbing Bian, Caohui Duan, Yongqin Xiong, Jiaji Lin, Haoxuan Lu, Linlin Deng, Ze Li, Mengting Wei, Jinhao Lyu, Ling Chen, Xin Lou

**Affiliations:** ^1^ Department of Radiology Chinese PLA General Hospital Beijing China; ^2^ School of Medical Imaging Guizhou Medical University Guiyang China; ^3^ Medical School of Chinese PLA Beijing China; ^4^ Department of Neurosurgery Chinese PLA General Hospital Beijing China

**Keywords:** 7‐T MRI, brain tumors, contrast‐enhanced MRI, ultrahigh field

## Abstract

**Aim:**

This study aimed to evaluate the diagnostic value of ultrahigh‐field magnetic resonance imaging (MRI) for brain tumors in clinical practice.

**Methods:**

Thirty patients with brain tumors underwent 7‐ and 3‐T MRI. The performance and diagnostic confidence of 7‐ and 3‐T MRI in the visualization of tumor details such as internal structure and feeding artery were evaluated by radiologists. Contrast‐enhanced region performance and tumor detail diagnostic confidence score (DCS) were calculated and compared between 7 and 3T using Wilcoxon rank sum test.

**Results:**

In 19 with obvious enhancement and 11 cases without obvious enhancement, 7‐ and 3‐T MRI showed similar performance. The tumors' internal structure and feeding artery were more clearly depicted by 7‐T MRI (62.2% and 54.4%, respectively) than by 3‐T MRI (2.2% and 6.7%, respectively). Furthermore, the mean DCSs of both internal structure and feeding artery were higher at 7T than at 3T (internal structure: 16.29 ± 9.67 vs. −5.79 ± 4.12, *p* = 0.028; feeding artery: 21.96 ± 6.93 vs. 4.46 ± 7.07, *p* = 0.028). The DCS was more significantly improved in the senior radiologist group.

**Conclusion:**

Better visualization of brain tumor details and higher tumor detail diagnostic confidence can be obtained with 7‐T MRI.

## Graphical abstract

Better visualization of brain tumors' internal structure (A, B) and tumors feeding artery (C, D) can be obtained at 7T than at 3T.

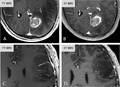

## 1 INTRODUCTION

Since its introduction, MR has become a powerful tool for investigating the neurological diseases (brain tumors, cerebrovascular diseases, etc.).^1^, ^2^, ^3^, ^4^ And since the 1990 s, more than 80 whole‐body human 7‐T magnetic resonance (MR) scanners have been installed worldwide, and the use of magnetic resonance imaging (MRI) in brain tumors diagnostic imaging is increasing steadily.^5^ Because of the linear relationship between signal‐to‐noise ratio (SNR) and static magnetic field strength, 7‐T MRI provides higher spatial resolution, SNR, and gray matter/white matter contrast, those can obtain richer information for clinical diagnosis and treatment.^6^, ^7^, ^8^, ^9^ As the static magnetic field strength increases, B0 and B1 inhomogeneities further increase, which can lead to spatially variable images.^10^ The impact of such changes on clinical practice needs to be evaluated.

Previous studies have shown that 7‐T MRI has certain advantages in the imaging of brain tumors. In brain metastases, 7‐T MRI increases the detection rate of metastatic lesions by 8% on contrast‐enhanced magnetization‐prepared rapid gradient echo (CE‐MPRAGE) images with the same dose of contrast medium.^11^ In pituitary adenomas, 7‐T CE‐T1‐weighted imaging can detect small lesions more accurately than 1.5‐T imaging.^12^ A study on gliomas reported that 7‐T CE‐MPRAGE images showed a higher contrast between the ring‐shaped enhancing tumor mass and the central necrosis of the tumors.^13^ However, all the above mentioned studies focused on a single disease, and the details of brain tumors at 7T were not described comprehensively.

Quantitative evaluation of brain tumor detail visualization at 7T is of great importance to clinical practice. When evaluating the diagnostic value of ultrahigh field strength MRI for brain tumors in clinical practice, it is important to determine whether 7‐T ultrahigh‐field MRI has more advantages than 3‐T MRI in conventional sequences, not only in terms of the details of a single category of brain tumors but also in terms of increase in field strength.

The aim of this study was to evaluate the degree of visualization of the internal structure and feeding artery of tumors and compare the diagnostic confidence of 7‐ and 3‐T MRI in brain tumors in order to determine the potential benefits of ultrahigh‐field MRI in clinical applications.

## 2 METHODS

### 2.1 Ethics

The trial was approved by our local research ethics committee and registered at ClinicalTrials.gov (NCT05287750), and written informed consent was obtained from the patients.

### 2.2 Patients

The inclusion criteria were adult patients (age ≥ 18 years) with suspected primary intracranial neoplasms (such as glioma or meningioma) or metastases and who were willing to undergo surgical treatment or puncture biopsy as well as preoperative MRI of the brain. Patients with contraindications to MRI scan, such as metal implants, dentures, claustrophobia, contrast agent allergy, and/or kidney failure, were excluded from the study. Demographic characteristics, including age and sex, are shown in Table 1. The study flowchart is shown in Figure S1.

**TABLE 1:** Clinical characteristics of the study participants.

Characteristic	Value
No. of patients	30
Age, year^a^	49.5 ± 11.74
Sex(male), *n* (%)	17 (56.7%)
Tumor location, *n* (%)	
Intracerebral	24 (80.0%)
Supratentorial	18 (60.0%)
Infratentorial	3 (10.0%)
Both	3 (10.0%)
Extracerebral	6 (20.0%)
Classification of tumor, *n* (%)	
Glioma	17 (56.7%)
Metastasis	4 (13.3%)
Meningioma	5 (16.7%)
Others^b^	4 (13.3%)

^a^ Data are means ± standard deviations.

^b^ One case of pineal germinoma, one case of medulloblastoma, and two cases of lymphoma. All tumor classifications were confirmed by pathology.

### 2.3 Data acquisition

The patients underwent imaging using a 7‐T MRI scanner Magnetom Terra with a 32‐channel head coil and a 3‐T MRI scanner Magnetom Skyra (both from Siemens Healthcare) with a 20‐channel head coil before and after gadolinium contrast agent administration (0.1 mmol/kg). The patients were first scanned with MPRAGE using a 7‐T MR scanner, followed by clinical sequence scans using a 3‐T MR scanner. The contrast agent was injected with an auto‐injector during scanning at 3T. To avoid changes in lesion enhancement performance with longer scanning time, the 7‐ and 3‐T CE‐MRI MPRAGE sequence scans were completed on the same day, with a time interval of 25 min after contrast agent injection.^14^, ^15^ The measurement sequence parameters were as follows: 7‐T MRI: repetition time (TR)/echo time (TE)/inversion time (TI), 2300/1.99/1050 ms; field of view (FOV), 224 × 224 mm; flip angle, 8°; in‐place resolution, 320 × 320; slice thickness, 0.7 mm; number of slices, 208; pixel bandwidth, 250 kHz; measurement duration, 5 min 14 s; 3‐T MRI: TR/TE/TI, 2300/2.98/900 ms; FOV, 256 × 256; flip angle, 9°; in‐place resolution, 224 × 210; slice thickness, 1.0 mm; number of slices, 176; pixel bandwidth, 235 kHz; measurement duration, 4 min 54 s.

### 2.4 Quantitative evaluation

**FIGURE 1:**
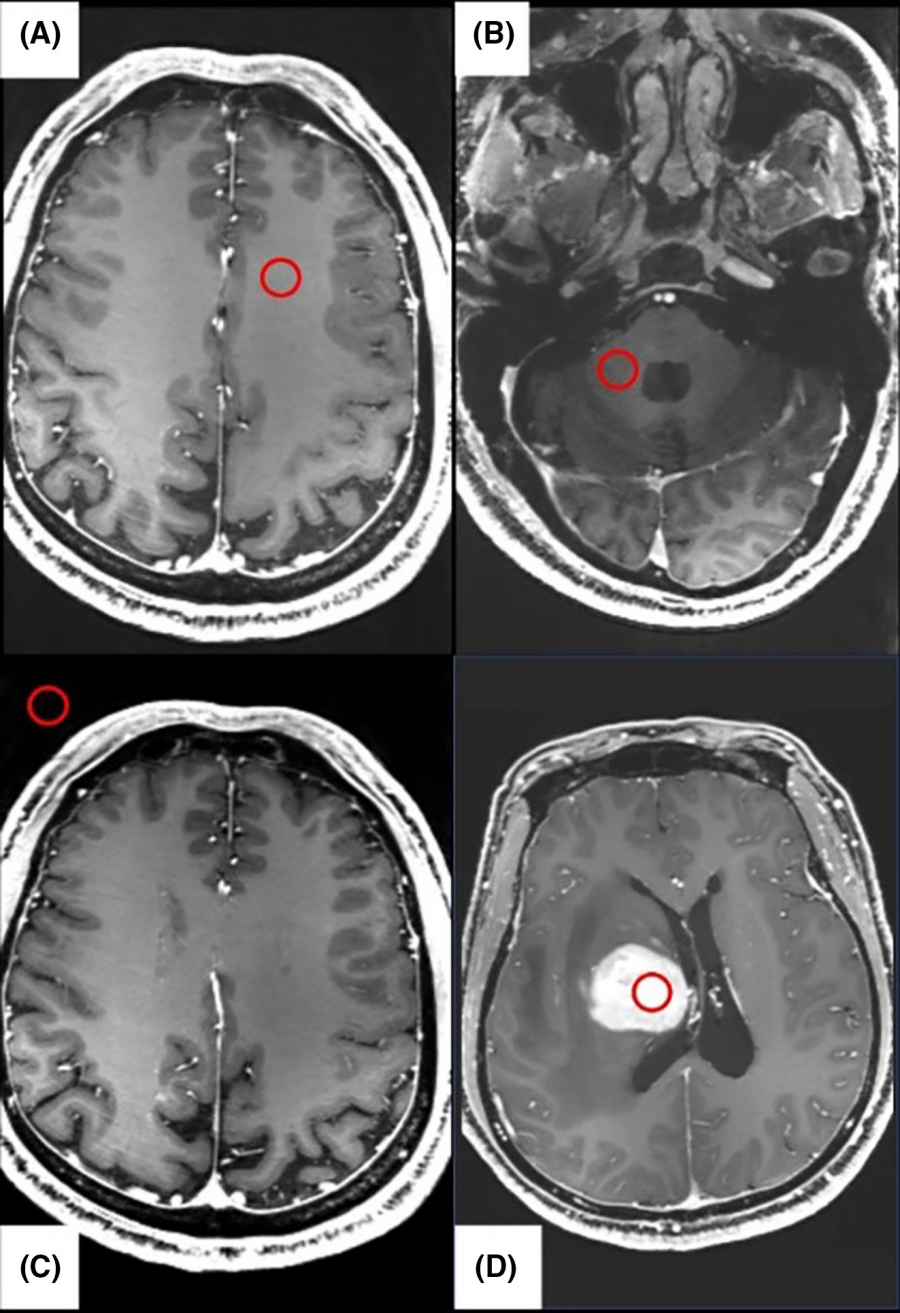
The regions of interest (ROI) were selected which were used to calculate the signal‐to‐noise (SNR) (red rings). The signal intensity of the white matter at the slice above the lateral ventricle and at the slice of the fourth ventricle was selected in (A) and (B) as S_NB_ and S_C_. The signal of the standard deviation (SD) of the background noise was selected in a region with no MR signal in (C) as SD_noise_. The signal intensity of lesion was selected in (D) as S_L_.

Quantitative assessment of image quality was performed by measuring the signal intensity in the region of interest (ROI). Two radiologists independently performed the measurement (K.‐C., 6‐year experience; C.X.‐L., 2‐year experience). The signal intensity of the white matter at the slice above the lateral ventricle and at the slice of the fourth ventricle was measured on the CE‐MPRAGE images (S_NB_ and S_C_), and the tumors' region of interest was selected to avoid areas of necrotic, cystic, and hemorrhagic components. The area of homogeneous signal was selected for measurement (S_L_). The standard deviation (SD) of the background noise was measured in a region with no MR signal (SD_noise_), the ROIs were illustrated in the Figure 1. For uneven brain MR signal due to coil‐related signal decrease in the posterior fossa, the brain white matter signal intensity (S_wm_) on CE‐MPRAGE images was the average of S_NB_ and S_C_. The SNR and contrast‐to‐noise ratio (CNR) of the lesions were calculated with the following equations: $\mathrm{SNR}=\frac{S_{L}}{{\mathrm{SD}}_{\text{noise}}}$, $\mathrm{CNR}=\frac{S_{L}-S_{\mathrm{wm}}}{{\mathrm{SD}}_{\text{noise}}}$.

### 2.5 Semi‐quantitative evaluation

Semi‐qualitative evaluation of the imaging quality in terms of image contrast, sharpness, and artifact was independently performed by two radiologists (Y.Q.‐X., 6‐year experience; K.‐C., 6‐year experience). Information about the examination field strength was removed before evaluation. Then, the sum from each item was calculated as the image quality score (IQS), and the IQS was divided into five grades, ranging from non‐diagnostic to excellent (see details in Table S1).

The evaluation of tumor detail score included two imaging criteria: internal structure and feeding artery (the evaluation criteria are presented in Table S2). Six radiologists (senior group: 3 radiologists with 5–6 years of experience; junior group: 3 radiologists with 2‐ or 3‐year experience) rated the tumor details of the internal structure and feeding artery at 7 and 3T. The evaluation of the images was performed independently, the order of evaluation of the two field strengths was randomized, and the readers were blinded to the field strengths.

The diagnostic confidence score (DCS) was developed as a 5‐point scale based on previous research, with minor adjustments in the tumor detail.^16^, ^17^ The DCS was calculated by assigning different weighted factors to the confidence levels (Table S2).

### 2.6 Statistical analysis

Continuous variables are presented as means ± SD. Categorical variables are expressed as ranges or medians (interquartile range), proportions, and percentages and were compared using Wilcoxon rank sum test. Inter‐observer agreement for MRI reading was assessed using intraclass correlation coefficient (ICC) and interpreted as follows: 0.0–0.5, poor reliability; 0.5–0.75, moderate reliability; 0.75–0.9, good reliability; 0.9–1.0, excellent reliability.^18^ All statistical calculations were performed using SPSS version 24.0 (IBM Corp.). A *p*‐value <0.05 was considered statistically different.

## 3 RESULTS

For brain tumors, the quality of 7‐T CE‐MPRAGE images was better than that of 3‐T MRI. The SNR and CNR results are presented in Table S3. For intra‐cerebral tumors, the SNR and CNR of the contrast‐enhanced images were significantly higher at 7T than at 3T (*p* < 0.05).

**FIGURE 2:**
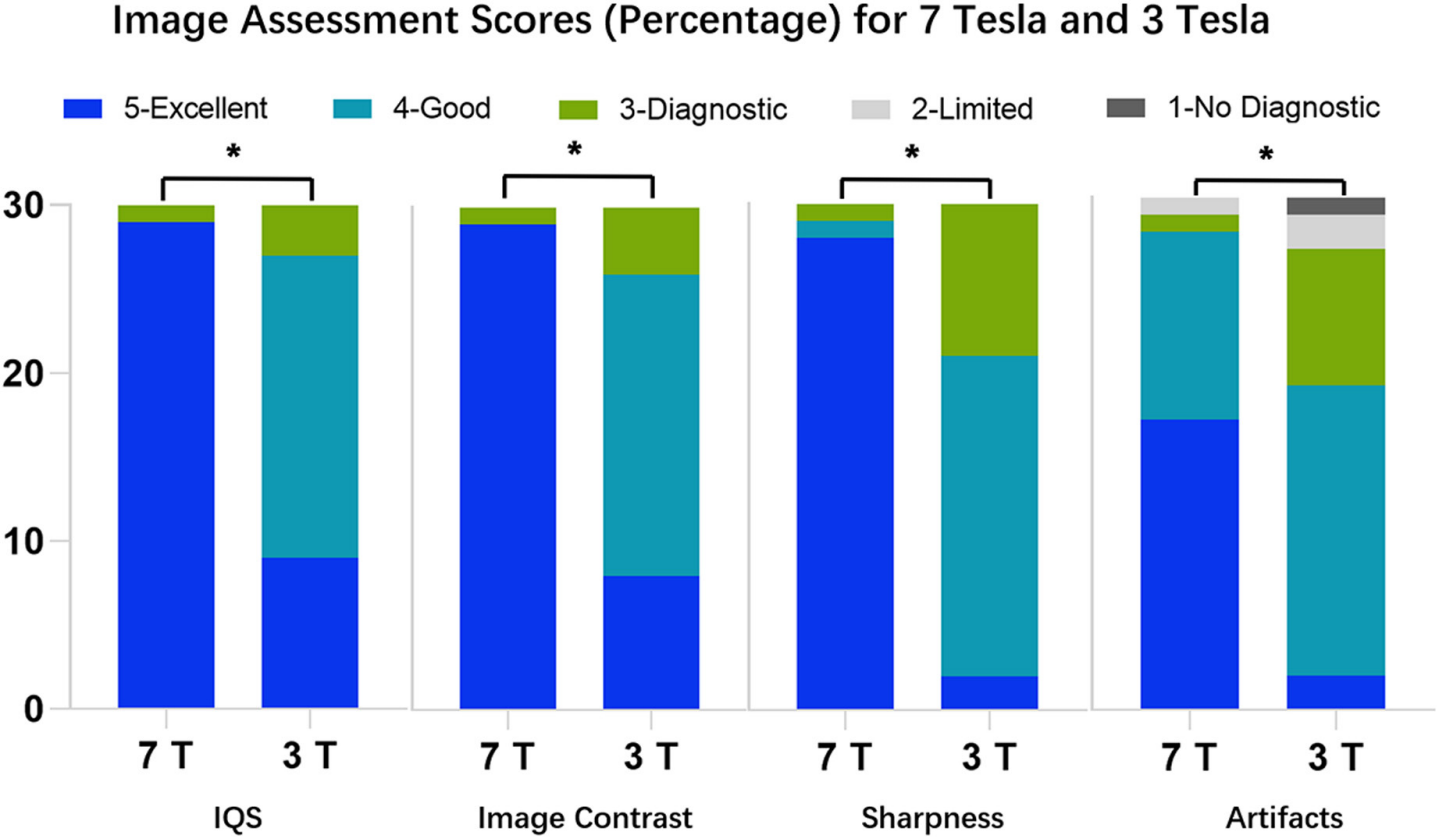
Percentage of the image quality score (IQS) in 7‐ and 3‐T contrast‐enhanced magnetization‐prepared rapid gradient echo images when evaluated by two radiologists in terms of overall IQS, image contrast, sharpness, and artifacts. Each color bar represents the percentage of cases with the same score. *Statistically different results, with *p* < 0.05.

The IQS was higher at 7T than at 3T, and evaluation of items such as contrast, sharpness, and artifact obtained better results at 7T than at 3T (Figure 2). All differences were statistically significant (Table S4).

**FIGURE 3:**
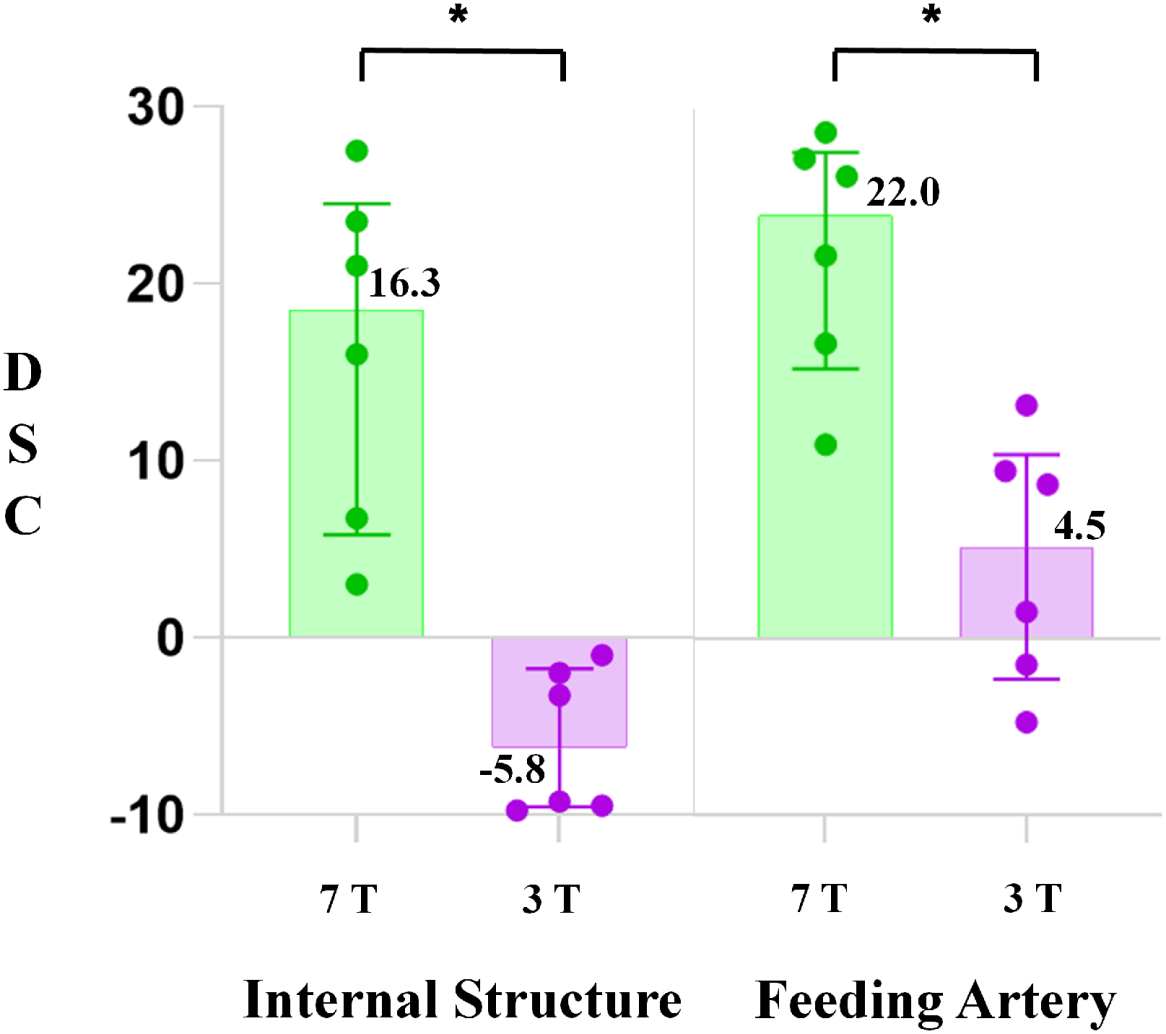
Mean diagnostic confidence scores (DCS) of the tumor's internal structure and feeding artery in 7‐ and 3‐T magnetic resonance images. The bar and dot plots represent the mean score of each category and exact data points, respectively; error bars represent the standard errors of mean. *Statistically different results, with *p* < 0.05.

Six radiologists evaluated 30 CE‐MPRAGE images, for a total of 180 evaluations at each field strength. In terms of tumor details, the tumors' internal structure was clearly visible in 112 evaluations (62.2%) at 7T and in four evaluations (2.2%) at 3T. The feeding artery was clearly visible in 98 evaluations (54.4%) at 7T and in 12 evaluations (6.7%) at 3T. The mean DSCs were significantly higher in both internal structure and feeding artery at 7T than at 3T for all radiologists (*p* < 0.05) (Figure 3). The average improvement was greater for the senior group (Table 2).

**TABLE 2:** Comparison of tumor details DCS at 7T and 3T.

	Junior Group			Senior Group			(Average, SD)	*p*‐value
	R1	R2	R3	R4	R5	R6		
Internal Structure								
7T	3.00	16.00	6.75	21.00	27.50	23.50	16.29 ± 9.67	0.028
3T	−9.75	−9.50	−2.00	−1.00	−9.25	−3.25	−5.79 ± 4.13	
*Δ* DCS	12.75	25.50	8.75	22.00	36.75	26.75		
Mean *Δ* DCS	15.67			28.50				
Feeding Artery								
7T	26.25	16.75	11.00	28.75	21.75	27.25	21.96 ± 6.93	0.028
3T	9.50	−4.75	−1.50	13.25	8.75	1.50	4.46 ± 7.07	
*Δ*DCS	16.75	21.50	12.50	15.50	13.00	25.75		
Mean *Δ*DCS	16.92			18.08				

Abbreviations: DCS, diagnostic confidence score; R1‐R3, radiologists with 2‐ or 3‐year diagnostic experience (junior group); R4‐R6, radiologists with 5‐ or 6‐year diagnostic experience (senior group); SD, standard deviations; ΔDCS, DCS at 7 TT minus DCS at 3 TT.

**FIGURE 4:**
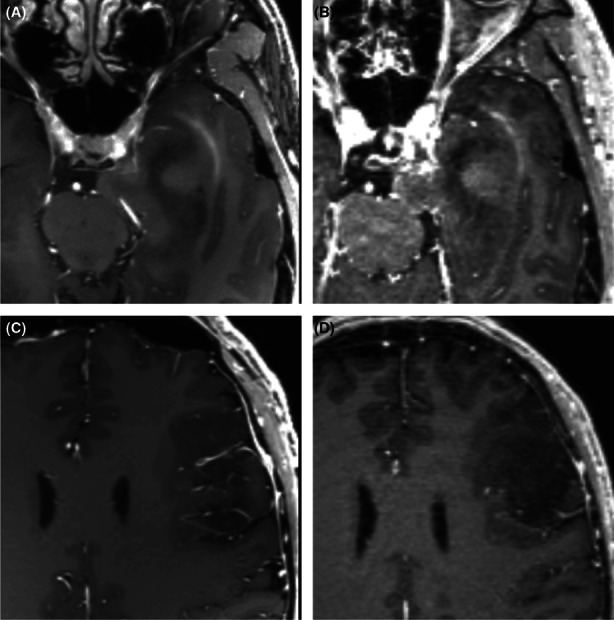
(A, B) Axial contrast‐enhanced magnetization‐prepared rapid gradient echo images at 7 (A) and 3T (B) of a 49‐year‐old male patient with low‐grade glioma, with the 7‐T image showing clearer lesion infiltration than the 3‐T image. (C, D) After contrast agent application at 7 (C) and 3T (D) in a 45‐year‐old male patient with low‐grade glioma, with the 7‐T image showing greater visibility of the feeding artery of the lesion than the 3‐T image.

**FIGURE 5:**
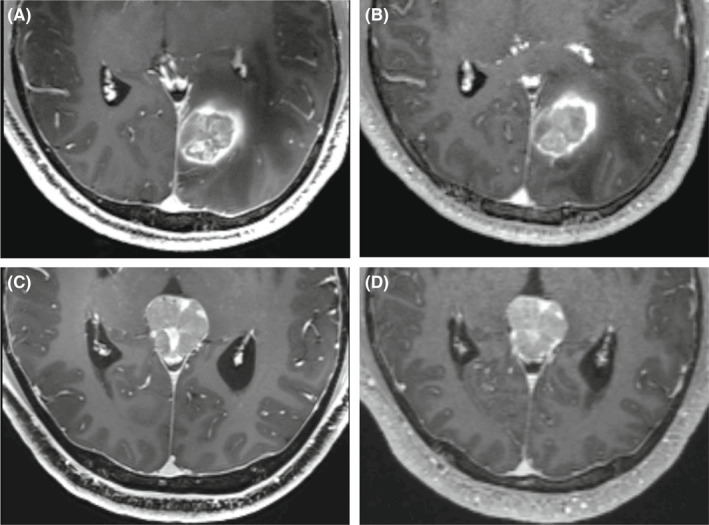
(A, B) Axial contrast‐enhanced magnetization‐prepared rapid gradient echo images at 7 (A) and 3T (B) of a 49‐year‐old male patient with metastasis, with the 7‐T image showing the internal structure of the lesion and the lesion infiltration more clearly than the 3‐T image. (C, D) After contrast agent application at 7 (C) and 3T (D) in a 54‐year‐old female patient with pineal germinoma, with the 7‐T image showing greater visibility of the internal structure of the lesion than the 3‐T image (C).

The types of tumor were high‐grade glioma (*n* = 7), lymphoma (*n* = 2), meningioma (*n* = 5), metastasis (*n* = 4), and pineal germinoma (*n* = 1), and these enhancing tumor masses were clearly visible in the 7‐ and 3‐T CE‐MPRAGE images. The region of enhancement of the tumor mass and the boundary of the enhancement region were similar at both field strengths. In 10 patients with low‐grade glioma and in 1 patient with medulloblastoma, the tumors showed no contrast enhancement or only mild enhancement in 3‐T CE‐MPRAGE; the tumors showed similar contrast enhancement performance at 7T, also with no contrast enhancement or only mild enhancement in the tumor mass region (Figures 4 and 5).

For image quality evaluation, two radiologists obtained moderate to excellent reliability in data measurement and evaluation (ICC > 0.6) (Table S5).

## 4 DISCUSSION

In this study, we performed quantitative and semi‐quantitative evaluation of CE‐MPRAGE images for brain tumors at 7 and 3T. We observed that the contrast enhancement for all brain tumors was similar on CE‐MPRAGE at both field strengths. However, 7T obtained higher image quality for brain tumors, better visualization of brain tumor details (intra‐tumoral structure and feeding artery), and higher tumor detail DCS. Better visualization of the intra‐tumoral structure and feeding artery may improve the accuracy of puncture biopsy and reduce bleeding during puncturing.

The SNR and CNR of the images increase as the field strength of the MR system increases in the same scan time. In a previous study, the SNR of 7‐T brain images without contrast medium was significantly better than that of 3‐T image, whereas the CNR was not significantly different.^16^ In our study, the SNR and CNR in 7‐T CE‐MPRAGE images were significantly higher than those in 3‐T images, which is due to the effect of contrast agent injection.^19^, ^20^ When the contrast agent reaches the tumor tissue, it shortens the longitudinal relaxation time of the tumor tissue and produces an MR signal different from that of normal brain tissue.^21^ The relaxation rate r_1_ of paramagnetic gadolinium‐based contrast agents described by the Solomon‐Bloembergen‐Morgan equation becomes smaller with increasing field strength.^22^ The r_1_ of gadolinium‐based contrast agents is lower at 7T than at 3T,^23^ which allows for shorter longitudinal relaxation time at 7T, thus providing stronger contrast with normal brain matter and greater visibility of the lesion. Our results are consistent with the findings of Noebauer‐Huhmann et al.^24^

The SNR and CNR of intra‐ and extra‐cerebral tumors did not differ. To our knowledge, the degrees of enhancement of different tumors differ. The CNR may be related to tumor classification or grade. For example, the degree of enhancement signal of a low‐grade glioma differs from that of a high‐grade glioma, which results in different corresponding CNRs on CE‐MPRAGE. Some studies on the sequences of susceptibility‐weighted imaging, MR spectroscopic imaging, and chemical exchange saturation transfer imaging at 7T showed the ability of 7‐T imaging in classifying different grades of glioma.^13^, ^25^, ^26^ We recognized that conventional sequence CE‐MPRAGE images at 7T may also be a convenient and more sensitive way to classify glioma grades.

For tumor detail DCS, all the evaluated items showed higher scores at 7T than at 3T with the same contrast agent dose. Clearer imaging of internal structures and feeding artery is important in clinical practice. For neurosurgeons, better visualization of the internal mass structure helps in obtaining correct tumor tissue with more confidence, thus improving the accuracy of puncture biopsy. Furthermore, they can avoid the feeding artery and thus reduce bleeding during puncturing as much as possible. In addition, imaging of tumor internal structures provides more insight into heterogeneous changes, which can improve the sensitivity in detecting glioma heterogeneity and thus explore the pathophysiological changes of glioma in a more in‐depth and comprehensive manner.^27^ This advantage facilitates the diagnosis of glioma heterogeneity and may provide an imaging exploration approach to drug resistance prediction.^28^ Furthermore, improvement of feeding artery visualization is helpful in differentiating benign and malignant tumors and grade diagnosis of glioma.^29^, ^30^ Our comparison results showed that the 7‐T CE‐MPRAGE images were better than the 3‐T images in visualizing tumor feeding artery, which may enhance the diagnostic efficacy for benign and malignant brain tumors and grade diagnosis of brain tumors.

In addition, we found that for all radiologists, the DCS increased in varying degrees at 7T, and the average increase in DCS in the senior group was higher than that in the junior group. Radiologists' confidence in diagnosing tumor details tends to increase with diagnostic experience; nevertheless, our findings show that the diagnostic confidence of radiologists can be improved by using 7‐T imaging.

Tumor boundary is an important imaging indicator in neurosurgery, as it determines the extent of tumor border resection and the maximum safe resection range of functional tumors, thus avoiding residual tumors.^31^, ^32^, ^33^, ^34^ For extra‐cerebral tumors such as meningioma, CE‐MPRAGE could depict the delineation of lesion margins. In contrast, intracranial tumors such as glioma, which are known to grow diffusely, show unclear boundary on imaging, and the degree of enhancement correlates with the histological grade of glioma. Contrast‐enhanced MR examination at 3T provides suggestions for diagnosis and neurosurgery. However, in our study, the enhancement performance was similar at 7 and 3T, and 7T did not seem to show better clinical cues than 3T in terms of tumor enhancement region and boundary. With the development of functional and metabolic imaging, combined with the high‐resolution features of 7T, the advanced sequences of 7‐T MRI show better potential for evaluating glioma borders.^25^, ^35^

Our study had some limitations. First, posterior cranial fossa‐related signals were less visible, as no other coils were used for imaging effectiveness display. Second, the number of cases of infratentorial tumors in this study was small; hence, more cases are needed to validate the performance and effectiveness of infratentorial tumors. Lastly, previous studies have shown similar contrast patterns with only half‐dose contrast agent at 7T and full dose at 3T. Because of the scan time and total dose of the contrast agent, we did not compare the effectiveness of 7T with half‐dose contrast agent and 3T with full‐dose contrast agent on tumor details (internal structure and feeding artery), and this need to be explored in the future.

In conclusion, the detection efficiency and contrast‐enhanced performance of 7‐ and 3‐T CE‐MPRAGE images on brain tumor lesion imaging were roughly similar, and 3‐T MRI remains the main diagnostic scanner in clinical practice. However, 7‐T CE‐MPRAGE images have demonstrated improved visualization of small structures and subtle brain tumor lesions within the same scan time, which may improve the accuracy of puncture biopsy and reduce bleeding during puncturing. Better visualization of tumor details had advantages in clinical practice, prognostic classification, and future diagnostic criteria.

## FUNDING INFORMATION

This work was supported by the National Natural Science Foundation of China (Nos.81825012, 81730048, and 82151309 to X.L).

## CONFLICTS OF INTEREST

The authors have no relevant financial or non‐financial interests to disclose. All authors declare that they have no known competing financial interests or personal relationships that could have appeared to influence the work reported in this article.

## Supporting information


Appendix S1.

## Data Availability

The data that support the findings of this study are available from the corresponding author upon reasonable request.
