# Supplementary material for: Evaluation of postcontrast images of intracranial tumors at 7T and 3T MRI: An intra‐individual comparison study
Source: CNS Neurosci Ther. 2022 Dec 5;29(2):559–65. doi: 10.1111/cns.14036 (PMC9873521; doi:10.1111/cns.14036)
Supplement: Supplementary file 1 — Appendix S1. [file CNS-29-559-s001.docx]

**Supplementary Material**

**Figure 1** The flowchart of patients.

**
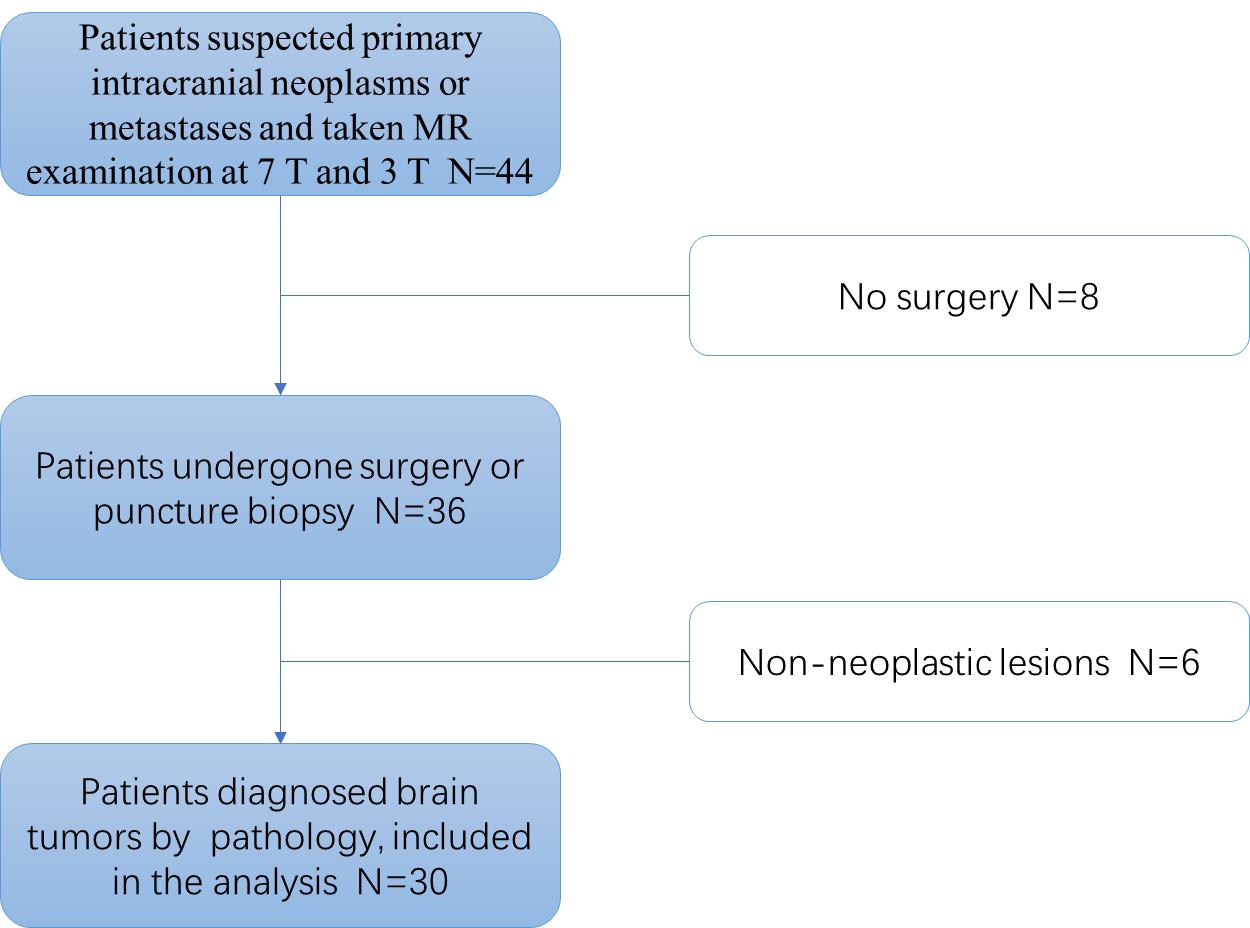
**

、

**Table 1** Scoring Criteria used for evaluation of contrast enhancement imaging at 7 T MRI and 3 T MRI.

| **IQS** | **Score** | **Contrast** | **Sharpness** | **Artifacts** |
| --- | --- | --- | --- | --- |
| Nondiagnostic  **0** | **0** | Some white matter, gray matter, basal ganglia and thalamus are separated on most images. | Some structures are not sharp on most images. | There are all three artifacts (aliasing, signal loss regions, or moving appearance) on all images. |
| Limited  **(1-3)** | **1** | Most white matter, gray matter, basal ganglia and thalamus are separated on some images. | Most structures are sharp on some images. | There are two types of artifacts among (aliasing, signal loss regions or moving appearance) on all images. |
| Diagnostic  **(4-6)** | **2** | Most white matter, gray matter, basal ganglia and thalamus are separated on most images. | Most structures are sharp on most images. | There is no aliasing, signal loss regions, or moving appearance on a few images. |
| Good  **(7-9)** | **3** | All white matter, gray matter, basal ganglia and thalamus are all separated on most images. | All structures are sharp on most images. | There is no aliasing, signal loss region, or moving appearance on most images. |
| Excellent  **(10-12)** | **4** | All white matter, gray matter, basal ganglia and thalamus are separated on all images. | All structures are sharp on all images. | There is no aliasing, signal loss region, or moving appearance on any image. |

**IQS**, Image Quality Score.

**Table 2** Tumor detail diagnostic confidence score rules and calculation formula.

|  | | | Diagnostic Confidence of Tumor Detail | |
| --- | --- | --- | --- | --- |
| Score | | Internal Structure | | Feeding Artery |
| 1 | | Definitely undisplay | | |
| 2 | | Quite likely undisplay | | |
| 3 | | Small chance of display | | |
| 4 | | Probably display | | |
| 5 | | Definitely display | | |
| DCS=1 × N_nd_ – 0.25 × N_qlu_ – 0.5 × N_ud_ – 0.25 × N_pd_ + 1 × N_dd_ | | | | |
| N_du_ | No. subjects with “Definitely undisplay” of this criterion | | | |
| N_qlu_ | No. subjects with “Quite likely undisplay” of this criterion | | | |
| N_ud_ | No. subjects with “uncertain display” of this criterion | | | |
| N_pd_ | No. subjects with “Probably display” of this criterion | | | |
| N_dd_ | No. subjects with “Definitely display” of this criterion | | | |

DCS indicates diagnostic confidence score.

**Table 3** The different of SNR and CNR at 7 T and 3 T, intracerebral and extracerebral at 7 T.

|  | 7 T | 3 T | *p*-Value | 7 T | | *p*-Value |
| --- | --- | --- | --- | --- | --- | --- |
|  |  |  |  | intracerebral | extracerebral |  |
| SNR | 234.6 (152.5, 331.6) | 78.2 (44.7, 113.9) | ＜0.001 | 234.6 (164.0, 380.4) | 204.2 (122.3, 349.8) | 0.756 |
| CNR | 109.6 (47.7, 212.9) | 31.6（-0.9, 49.3） | ＜0.001 | 120.0 (22.1, 245.1) | 85.2 (62.8, 179.8) | 0.917 |

Values represent median (interquartile range). SNR, signal-to-noise ratio. CNR, contrast-to-noise ratio.

**Table 4** Comparison of image quality score at 7 Tesla and 3 Tesla

|  | **7 T** | **3 T** | ***p*-Value** |
| --- | --- | --- | --- |
|  | (n=30) | (n=30) |  |
| **Contrast** | 4 (4, 4) | 3 (3, 4) | ＜0.001 |
| **Sharpness** | 4 (4, 4) | 3 (2, 3) | ＜0.001 |
| **Artifacts** | 4 (3, 4) | 3 (2, 3) | ＜0.001 |
| **Image Quality Score** | 12 (11,12) | 9 (7.75, 9) | ＜0.001 |

Values represent median (interquartile range).

**Table 5** List of intraclass correlation efficient (ICC).

| **Project** | **ICC (95% CI)** | ***p*** -**Value** |
| --- | --- | --- |
| **ROI intensity (all value)** | 0.940 (0.925, 0.953) | ＜0.001 |
| **ROI intensity (in 7 T MRI)** | 0.937 (0.917, 0.953) | ＜0.001 |
| **ROI intensity (in 3 T MRI)** | 0.906 (0.816, 0.937) | ＜0.001 |
| **Imaging Quality Score（all value）** | 0.672 (0.540, 0.771) | ＜0.001 |
| **Imaging Quality Score (at 7 T MRI)** | 0.839 (0.733, 0.902) | ＜0.001 |
| **Imaging Quality Score (at 3 T MRI)** | 0.707 (0.586, 0.797) | ＜0.001 |

This coefficient was interpreted as excellent (0.90–1.00), good (0.75–0.90), moderate (0.50–0.75), and poor (＜0.50).
